# Supplementary figures and images for: Repression of ZCT1, ZCT2 and ZCT3 affects expression of terpenoid indole alkaloid biosynthetic and regulatory genes
Source: PeerJ. 2021 Jul 2;9:e11624. doi: 10.7717/peerj.11624 (PMC8256811; doi:10.7717/peerj.11624)

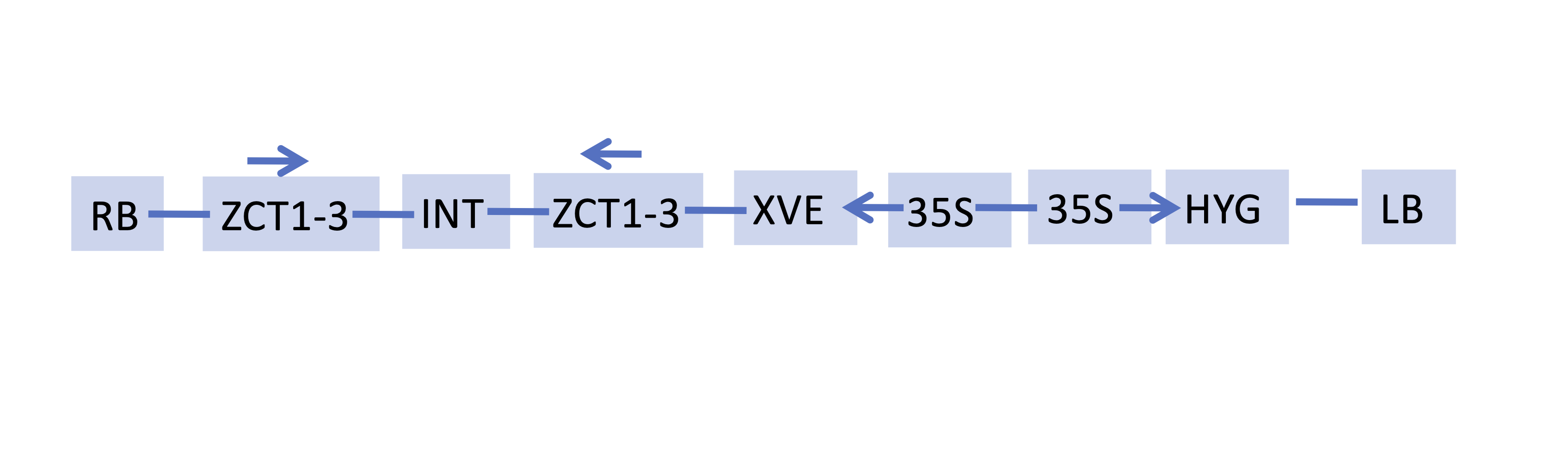

Supplement: Supplemental Information 2 — The orientations of the T-DNA right border (RB), fragments of the ZCT1, ZCT2 and ZCT3 genes (ZCT1-3), spacer present in the original pOpOff2(Hyg) RNAi vector (INT), XVE regulatory sequences (XVE), Cauliflower mosaic virus 35S promoter sequences (35S), hygromycin resistance gene (HYG) and T-DNA left border (LB) are indicated. [file peerj-09-11624-s002.png]
